# Herbal medicine *Siho-sogan-san* for functional dyspepsia: A protocol for a systematic review and meta-analysis

**DOI:** 10.1097/MD.0000000000022358

**Published:** 2020-09-25

**Authors:** Na-Yeon Ha, Hae-in Jeong, Ha-nul Lee, Seok-Jae Ko, Jae-Woo Park, Jinsung Kim

**Affiliations:** Department of Gastroenterology, College of Korean Medicine, Kyung Hee University, Seoul, Republic of Korea.

**Keywords:** functional dyspepsia, protocol, randomized controlled trials, *Siho-sogan-san*, systematic review

## Abstract

**Background:**

Functional dyspepsia (FD) is characterized by persistent and recurrent dyspeptic symptoms, such as postprandial fullness and epigastric pain. Although it is rarely severe or life-threatening, it can degrade the quality of life and cause social and economic issues. As symptoms often persist despite the treatment with conventional Western medicine, herbal medicine can be considered as an alternative for treating FD. *Siho-sogan-san* (SHS) is a traditional herbal formula prescribed for dyspepsia for hundreds of years. This protocol for a systematic review was designed to evaluate the safety and efficacy of SHS for the treatment of FD through a meta-analysis.

**Methods:**

Studies will be searched from the following electronic databases up to March 2020: Embase, MEDLINE (via PubMED), Cochrane Central Register of Controlled Trials, Allied and Complementary Medicine Database, Korean Medical Database, KoreaMed, Korean Studies Information Service System, National Digital Science Library, Oriental Medicine Advanced Searching Integrated System, China National Knowledge Infrastructure Database, and Citation Information by Nii. Randomized controlled trials of SHS and herb-added SHS for treating FD will be selected in this review. The control groups of no-treatment, placebo, and conventional Western medicine will be compared with SHS for its efficacy. The synergetic effect of SHS with Western medicine will also be analyzed in comparison with conventional Western medicine alone. Two independent reviewers will collect the data and assess the risk of bias in individual studies. The total clinical effectiveness rate will be synthesized and evaluated as primary outcome.

**Results:**

This systematic review will present an adequate clinical evidence of SHS for the treatment of FD based on specific parameters, including dyspepsia-related symptoms, gastric emptying, and adverse events.

**Conclusion:**

This study will provide evidence for the safety and efficacy of SHS for the treatment of patients with FD.

**Review Registry Unique Identifying Number:**

reviewregistry952.

## 1 Introduction

In general, patients with functional dyspepsia (FD) complain of postprandial fullness, early satiety, epigastric pain, and epigastric burning.^[1]^ According to the Rome IV diagnostic criteria, FD is diagnosed based on symptoms alone and does not require any evidence of systemic problems.^[2]^ The mechanisms underlying FD are unclear. FD can affect the patient's quality of life and accounts for 14% to 66% of the medical care burden in several countries.^[3]^ It is divided into the following 2 subgroups depending on the symptoms: postprandial distress syndrome and epigastric pain syndrome. Several underlying pathophysiologic mechanisms, such as impaired gastric motility, visceral hypersensitivity, and psychological factors, have been reported.^[4,5]^ Although *Helicobacter pylori* eradication, proton pump inhibitor therapy, and other additional treatment have been proposed to manage patients with FD, most of symptoms usually persist and recur. It explains in part why FD patients seek complementary and alternative medicine, including herbal medicine, despite their limited evidence.^[6]^

*Siho-sogan-san* (SHS) is a traditional herbal prescription, composed of 7 herbs, that has been widely used to improve dyspeptic symptoms similar with FD for hundreds of years. A review of meta-analysis on 13 randomized controlled trials (RCTs) showed that traditional Chinese medicine (TCM) was more effective in the treatment of FD with liver–stomach disharmony syndrome compared to prokinetics, without serious adverse effects. Among selected studies, *Radix Paeoniae*, *Radix Bupleuri*, *Radix Glycyrrhizae*, and *Rhizoma Cyperi* were frequently included and prescribed in the formula, in that order. In addition, these herbs make up SHS as well, functioning in soothing the liver and regulating *qi*.^[7]^ An animal study showed that *Rhizoma Cyperi* had an effect to improve gastric emptying and reduce gastric damage.^[8]^ Saikosaponin, a main component of *Radix Bupleuri*, has anti-inflammatory and antidepressant-like effects.^[9,10]^ From the perspective that there are some relations between functional gastrointestinal disorders (FGIDs) and psychosocial factors, such as anxiety disorder and depression, it is expected that SHS may be effective and safe for treating FD.^[11]^

In a previous review of meta-analysis, SHS showed higher efficacy and safety than prokinetic drugs. However, this analysis was limited in quality of included studies, database resources, type of comparators, and outcome parameters.^[12]^ Therefore, the aim of this review is to provide proper evidence to support the clinical usage of SHS for the treatment of FD, through the procedure of searching and synthesizing RCTs of SHS on FD systematically.

## 2 Methods

### 2.1 Eligibility criteria

#### 2.1.1 Types of studies

RCTs and quasi-RCTs will be included in this systematic review, whereas animal or cell research, non-RCTs, and review articles will be excluded.

#### 2.1.2 Types of participants

Patients diagnosed with FD using the Rome criteria will be included, regardless of the sex, age, and race. The latest revised version, that is, version IV, of the Rome criteria was established in 2016 and is used as the standard for diagnosing FGIDs, including FD. Two reviewers (HJ and HL) will independently classify the study by the time of its publication based on 1991 and apply similar criteria as the Rome criteria upon the inter-agreement eligibility (e.g., persistent dyspeptic symptoms without any structural defects). Although secondary symptoms caused by other diseases should be excluded, some patients will be selected in this review because the possibility of accompanying morbidity of FD with gastroesophageal reflux disease or irritable bowel syndrome was raised in the Rome IV criteria.

#### 2.1.3 Types of interventions

Studies on SHS, including modified SHS (herb-added formula) alone or in combination with Western medicine, will be selected. The following comparisons will be made with SHS: no other treatment, placebo (indistinguishable from real SHS), and Western drugs, including prokinetics and antidepressants. If possible, this review will also analyze the efficacy of SHS in combination with other Western drugs for the treatment of FD compared to conventional Western medicine alone.

#### 2.1.4 Types of outcome measures

The total clinical effective rate will be analyzed as primary outcome. Secondary outcomes will include dyspepsia-related symptom score, gastric emptying, quality of life, adverse events, and so on.

### 2.2 Search studies

#### 2.2.1 Database resources

The following electronic databases will be included in this study, regardless of the language, from its inception to March 2020:

1. Embase
2. MEDLINE (via PubMED)
3. Cochrane Central Register of Controlled Trials
4. Allied and Complementary Medicine Database
5. Korean Medical Database
6. KoreaMed
7. Korean Studies Information Service System
8. National Digital Science Library
9. Oriental Medicine Advanced Searching Integrated System
10. China National Knowledge Infrastructure Database
11. Citation Information by Nii

In addition, appropriate data from Clinical Research Information Service and ClinicalTrials.gov will be included.

#### 2.2.2 Search strategy

We will apply search strategies, including the terms of disease part (e.g., disturbance, illness, indigestion, and gut) and intervention part (e.g., herb, *Siho, Saiko, and Chaihu*) according to each database format (e.g., shown in Table 1 for PubMed).

**Table 1:**
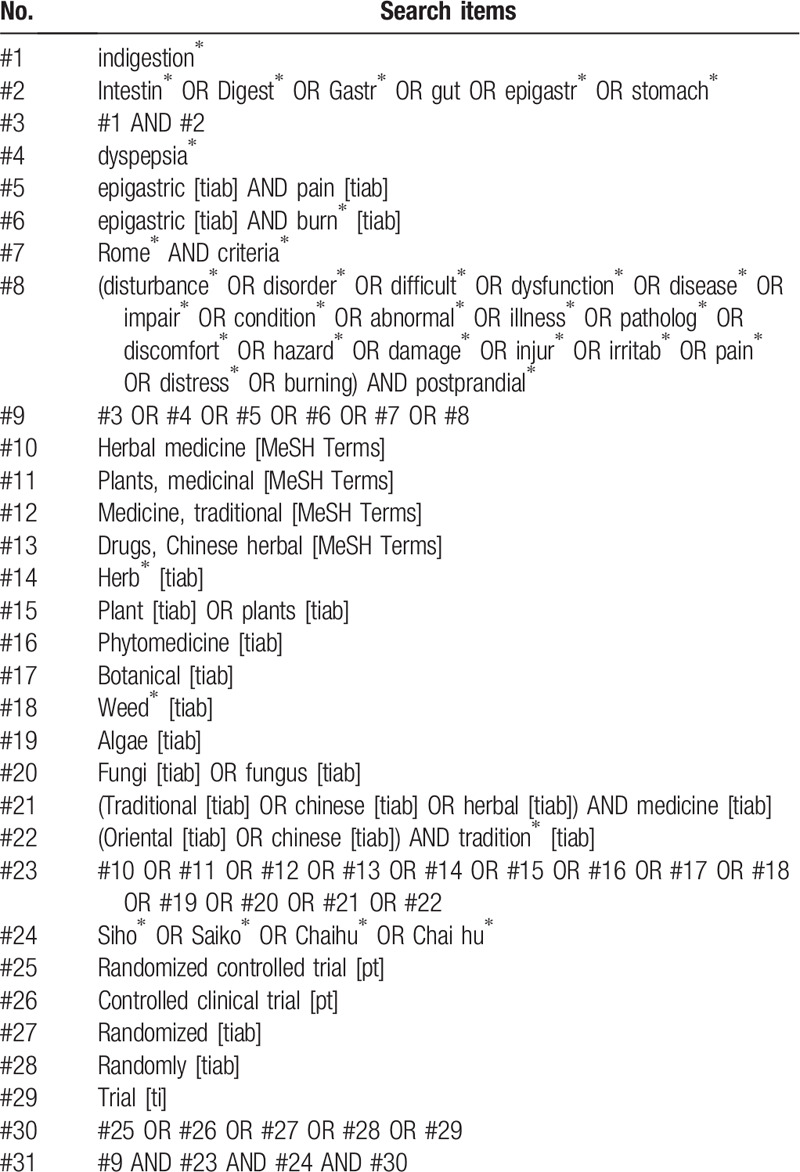
Search strategy used in PubMed.

### 2.3 Data collection and assessment

#### 2.3.1 Study selection

**Figure 1:**
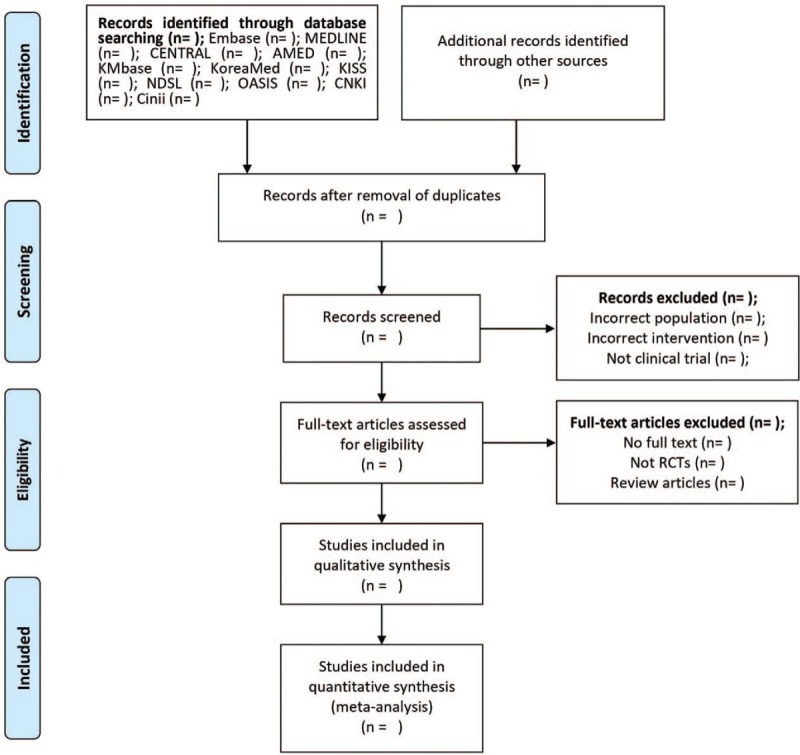
Flow chart of the search process. AMED = Allied and Complementary Medicine Database, CENTRAL = Cochrane Central Register of Controlled Trials, Cinii = Citation Information by Nii, CNKI = China National Knowledge Infrastructure Database, KISS = Korean Studies Information Service System, KMbase = Korean Medical Database, NDSL = National Digital Science Library, OASIS = Oriental Medicine Advanced Searching Integrated System, RCTs = randomized controlled trials.

Two reviewers (HJ and HL), well-educated in the search process, will independently select relevant articles, screening the titles and abstracts and excluding duplicates or inappropriate studies. The articles will be uploaded and arranged by Endnote X8 (Clarivate Analytics). For screening, the investigators (HJ and HL) will independently review the full text of articles to select suitable ones. Details of the study selection process are presented in a Preferred Reporting Items for Systematic Review and Meta-analysis flow diagram (Fig. 1). Disagreement between 2 reviewers will be resolved by adjustments of an arbiter (N-YH).

#### 2.3.2 Data extraction

Two authors (HJ and HL) will independently extract related data with this review and fill the standard data extraction form. It will include information, such as the first author, publication year, study design, intervention, comparator, administration period, outcomes, and adverse events. Differences between the reviewers will be discussed and resolved by the arbiter (N-YH).

#### 2.3.3 Assessment of the risk of bias in included studies

The risk of bias will be assessed independently by 2 investigators (HJ and HL) according to the Cochrane Collaboration's tool. The following items will be included: random sequence generation (selection bias), allocation concealment (selection bias), blinding of participants and personnel (performance bias), blinding of outcome assessment (detection bias), incomplete outcome data (attrition bias), selective outcome reporting (reporting bias), and other bias. Each assessment will be divided into 3 stages: low, unclear, and high. The reviewers will discuss all disagreements, and the arbiter (N-YH) will intervene the problem and obtain a consensus.

### 2.4 Data analysis

#### 2.4.1 Statistical analysis

The outcomes will be reported as the mean difference with 95% confidence interval (CI) for continuous data and risk ratio or odds ratio with 95% CI for categorical data.

#### 2.4.2 Handling missing data

We will contact the authors of original studies by sending an email to request for missing data. The outcomes will be assessed based on the intent-to-treat analysis.

#### 2.4.3 Assessment of heterogeneity

Random-effects models will be included in the meta-analysis. Heterogeneity in the included studies will be assessed by the *I*-squared statistic and chi-squared (*χ*^*2*^) test (*I*^*2*^ ≥ 50% and *P* value < .1 mean substantial heterogeneity).

#### 2.4.4 Data synthesis

The Review Manager program (version 5.3. Copenhagen: The Nordic Cochrane Centre, The Cochrane Collaboration, 2014) will be used for data synthesis. To evaluate the efficacy of SHS, the outcomes will be synthesized and compared with several types of interventions, such as no-treatment, placebo, and conventional Western medicine. If sufficient studies are included, the effect of SHS combined with conventional Western medicine versus Western medicine alone will also be measured to confirm the synergetic effect of the combination therapy.

#### 2.4.5 Subgroup analysis

If it is necessary to investigate the causes of high heterogeneity in included studies, we will conduct a subgroup analysis of the following items: pattern identification of TCM, types of herbal medicine, administration period, and so on.

#### 2.4.6 Assessment of publication bias

If there are more than 10 studies for the analysis, a funnel plot will be presented to evaluate the publication bias and assess small study effects.

#### 2.4.7 Sensitivity analysis

“The Consolidated Standards of Reporting Trials and Extension for Herbal Interventions” will be applied for assessing the quality of reports. We will conduct a sensitivity analysis to assess the robustness of the findings in the meta-analysis.

#### 2.4.8 Grading the quality of evidence

“The Grading of Recommendations Assessment, Development and Evaluation” will be used for assessing the quality of evidence.

### 2.5 Ethics and dissemination

In this study, ethical approval is not required because the included data are based on previously reported articles, and identifying information of participants will not be revealed. Results of this systematic review will be submitted for publication in a peer-reviewed journal and disseminated electronically and in print.

## 3 Discussion

*Siho-sogan-san* (SHS in Korea, *Chaihu-Shugan-San* [CSS] in China, and *Saiko-sokan-to* [SST] in Japan) is a traditional herbal formula that has been prescribed to alleviate dyspeptic symptoms for hundreds of years.^[12]^ SHS includes the following 7 crude herbs: *Radix Bupleuri, Aurantii nobilis Pericarpium, Rhizoma Cnidii, Rhizoma Cyperi, Radix Paeoniae, Poncirus trifoliata Rafinesque,* and *Radix Glycyrrhizae*.^[13]^ Animal studies showed that administration of CSS promoted gastric emptying and intestinal transit.^[14]^

According to TCM theories, SHS has been applied to patients with various symptoms of the syndrome of liver–stomach disharmony due to liver–*qi* stagnation, closely related to emotional instability. The liver–stomach disharmony can be induced by stagnation of *qi* due to depression. Thus, soothing the liver, regulating the depressed *qi* and stomach, and finally, relieving pain is the treatment principle of SHS to improve gastrointestinal dysfunction.^[12]^ Through a previous review of meta-analysis for 10 RCTs, the effectiveness and safety of CSS for the treatment of depression has been confirmed.^[15]^ In an animal model study, CSS had an antidepressant effect on the repression of anger and distress.^[13]^ In another animal model, CSS regulated hyperactivity of the hypothalamic–pituitary–adrenal axis in rats resulting from chronic stress and exerted antidepressive effects.^[16]^

The review of meta-analysis for 22 RCTs of modified *Chaihu Shugan* powder (MCSP) for treating FD showed that MCSP, used both alone and together with prokinetic drugs, was more effective than prokinetic drugs alone, without inducing any serious adverse events.^[12]^ There were some limitations to present adequate clinical evidence for proving MCSP's effectiveness. Above all, a high risk of bias and poor methodology were observed in the included studies. Language and databased publications were limited to only English and Chinese, and only total effective rates were evaluated and compared as outcome. Thus, in this systematic review of meta-analysis, we will use various database resources, including from Korea and Japan, in order to research the evidence of herbal medicine comprehensively. Moreover, herb-added formulas of SHS will be included and analyzed as the treatment group with no change in the primary prescription principles, reflecting variously modified form from the original composition. This study will update the safety and efficacy of SHS in monotherapy or in combination with Western medicine, including not only prokinetic drugs also acid-suppressive agents, antidepressants, based on the recent RCTs. The comparison groups will be set to no-treatment, placebo, and conventional Western medicine alone to provide clinical evidence. In case of enough studies to be analyzed, quality of life and objective parameters, such as gastric emptying, will be synthesized and evaluated for assessing outcomes. The results will be used to identify the effectiveness of SHS to improve other factors in addition to subjective dyspepsia symptoms, and conversely, to determine the pathophysiology of FD. We anticipate that this review will provide clinical information for the usage of SHS in detail.

In this protocol for a systematic review, ethics approval is not required because it is based on information gathered by searching already reported articles and no additional primary data collected. Results of this systematic review will be published in a peer-reviewed journal and provided as electronic dissemination.

## Acknowledgments

This study was supported by a grant through the project “Development of Korean medicine clinical practice guidelines” of the Guideline Center for Korean Medicine, National Institute for Korean Medicine Development.

## Author contributions

**Conceptualization:** Na-Yeon Ha, Hae-in Jeong.

**Data curation:** Na-Yeon Ha, Hae-in Jeong, Ha-nul Lee, Seok-Jae Ko, Jae-Woo Park, Jinsung Kim.

**Formal analysis:** Jinsung Kim.

**Investigation:** Na-Yeon Ha, Hae-in Jeong, Ha-nul Lee.

**Methodology:** Seok-Jae Ko, Jae-Woo Park, Jinsung Kim.

**Resources:** Jinsung Kim.

**Writing – original draft:** Na-Yeon Ha.

**Writing – review & editing:** Jinsung Kim.
